# Characterization and in vitro data of antibody drug conjugates (ADCs) derived from heterotrifunctional linker designed for the site-specific preparation of dual ADCs

**DOI:** 10.1016/j.dib.2018.11.005

**Published:** 2018-11-06

**Authors:** Amit Kumar, Krista Kinneer, Luke Masterson, Ebele Ezeadi, Philip Howard, Herren Wu, Changshou Gao, Nazzareno Dimasi

**Affiliations:** a Antibody Discovery and Protein Engineering, MedImmune, Gaithersburg, Maryland 20878, United States; b Oncology Research, MedImmune, Gaithersburg, Maryland 20878, United States; c Spirogen Ltd., QMB Innovation Centre, London, United Kingdom

## Abstract

Experimental procedures and ^1^H and ^13^C NMR of the heterotrifunctional linker used for preparation of dual drug conjugates and PBD payload are included. Procedure for carrying preparation of antibody linker conjugate via thiol maleimide conjugation and antibody drug conjugates (ADCs) using copper assisted click reaction and oxime ligation, their cell viability assay and western blotting procedures of the resultant conjugates are detailed. Also, reduced mass spectroscopy results and in vitro cytotoxicity of antibody drug conjugates used in this article are shown.

**Specifications table**

**Table:** 

Subject area	*Chemistry, biology*
More specific subject area	*Antibody Drug Conjugates*
Type of data	*Experimental procedures, NMR, Mass Spectrometry*
How data was acquired	*NMR, Mass spectrometry*
Data format	*Analyzed*
Experimental factors	*None*
Experimental features	NMR, Reduced mass spectroscopy
Data source location	*Gaithersburg, Maryland*
Data accessibility	*Data is with this article*
Related research article	*Levengood, M.R., et al., Orthogonal Cysteine Protection Enables Homogeneous Multi*‐*Drug Antibody–Drug Conjugates. Angewandte Chemie (International Ed. in English), 2017. 56(3): p. 733–737.*

**Value of the data**

In the data presented here we describe the synthesis of a heterotrifunctional linker serving as a flexible platform for the preparation of dual-cytotoxic drug conjugates in a site-specific manner [1]. As a proof of concept, we detail the preparation and evaluation of dual drug ADCs carrying monomethyl auristain E (MMAE) and pyrrolobenzodiazepine dimer (PBD).

## 1 Data

Experimental procedures and NMR of the linker used for preparation of dual drug conjugates and PBD payload are included. Procedure for carrying preparation of antibody drug conjugates (ADCs), their cell viability assay and western blotting procedures are detailed. Also, reduced mass spectroscopy results of antibody drug conjugates used in this article are listed.

## 2 Experimental design, materials, and methods

### 2.1 General information

All reagents were purchased through VWR or Sigma Aldrich and were used without further purification. ^1^H and ^13^C NMR spectra were obtained on a Bruker Ascend 400 spectrometer. Coupling constants are quoted in hertz (Hz). Mass Spectrometry was obtained using a Waters Acquity UPLC LCMS. O-vc-PAB-MMAE was purchased from Concortis.

### 2.2 Synthesis of linker 1

**Figure:**
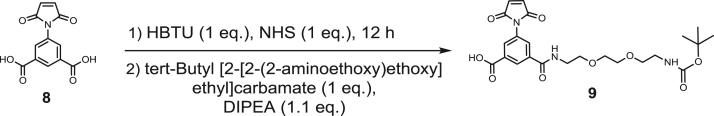

Synthesis of 3-((2,2-dimethyl-4-oxo-3,8,11-trioxa-5-azatridecan-13-yl)carbamoyl)-5-(2,5-dioxo-2,5-dihydro-1H-pyrrol-1-yl)benzoic acid. A solution of compound **8** (10 g, 28.2 mmol) in DMF (25 mL) was treated with HBTU (7.3 g, 19.1 mmol) and NHS (2.2 g, 19.1 mmol). The solution was stirred overnight at room temperature followed by addition of tert-Butyl [2-[2-(2-aminoethoxy)ethoxy]ethyl]carbamate (4.8 g, 19.1 mmol) and DIPEA (3.7 mL, 21.0 mmol) at 0 °C. The resulting mixture was stirred at room temperature for 1 h at 0 °C followed by removal of solvent under reduced pressure. The resulting crude mixture was purified by reverse phase chromatography to yield **9** as a white solid (3.3 g, 36%). Less than 10% of disubstituted product is observed during the reaction and can easily be removed via chromatography. ^1^H NMR (400 MHz, Methanol-*d4*) δ 8.50 (s, 1H), 8.21 (s, 1H), 8.10 (s, 1H), 7.04 (s, 2H), 3.78-3.59 (m, 8H), 3.58-3.49 (m, 2H), 3.258-3.19 (m, 2H), 1.42 (s, 9H). ^13^C NMR (101 MHz, Methanol-*d4*) δ 27.4, 39.7, 69.1, 69.7, 69.9, 78.7, 126.9, 128.9, 129.7, 134.3, 135.6, 166.6, 166.9, 169.4. MS (ESI) m/z calculated for C_23_H_29_N_3_O_9_: 491.2; found: 492.2 [M+H]^+^.

**Figure:**
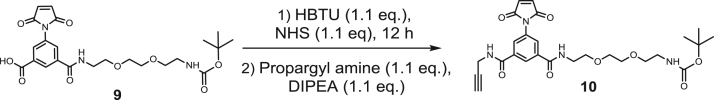

Synthesis of tert-butyl (2-(2-(2-(3-(2,5-dioxo-2,5-dihydro-1H-pyrrol-1-yl)-5-(prop-2-yn-1-ylcarbamoyl)benzamido)ethoxy)ethoxy)ethyl)carbamate. A solution of compound 9 (3 g, 6.1 mmol) in DMF (5 mL) was treated with HBTU (2.5 g, 6.7 mmol) and NHS (0.78 g, 6.7 mmol). The solution was stirred overnight at room temperature followed by addition of propargyl amine (0.47 mL, 7.3 mmol) and DIPEA (1.3 mL, 7.3 mmol). The resulting mixture was stirred at room temperature for 1 h followed by removal of solvent under reduced pressure. The resulting crude mixture was purified by reverse phase chromatography to yield **10** as a white solid (2.2 g, 69%). ^1^H NMR (400 MHz, Methanol-*d4*) δ 8.18 (s, 1H), 7.87 (s, 2H), 6.90 (s, 2H), 4.07 (s, 2H), 3.57-3.44 (m, 10H), 3.25-3.10 (m, 2H), 2.60 (s, 1H), 1.28 (s, 9H). ^13^C NMR (101 MHz, Methanol-*d4*) δ 27.5, 28.9, 39.8, 69.1, 69.7, 71.3, 78.7, 79.4, 124.9, 127.7, 127.9, 132.4, 135.1, 135.7, 156.9, 166.3, 166.8, 169.5. MS (ESI) m/z calculated for C_26_H_32_N_4_O_8_: 528.2; found: 529.2[M+H]^+^.

**Figure:**
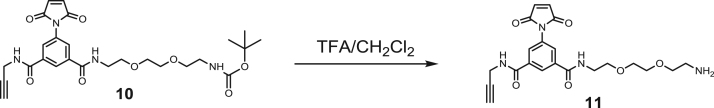

Synthesis of N1-(2-(2-(2-aminoethoxy)ethoxy)ethyl)-5-(2,5-dioxo-2,5-dihydro-1H-pyrrol-1-yl)-N3-(prop-2-yn-1-yl)isophthalamide 5. To a cooled solution of 10 (2.0 g, 3.8 mmol) in dichloromethane (2 mL) was added TFA (2 mL) in a dropwise manner. The resulting solution was stirred at room temperature for 2 h followed by removal of solvent under reduced pressure. The resulting crude mixture was purified by reverse phase chromatography to yield **11** as a white solid (1.5 g, 93%). ^1^H NMR (400 MHz, Methanol-*d4*) δ 8.32 (s, 1H), 8.00 (s, 2H), 6.98 (s, 2H), 4.02 (s, 2H), 3.78-3.59 (m, 8H), 3.58-3.49 (m, 2H), 2.54 (s, 2H). ^13^C NMR (101 MHz, Methanol-*d4*) δ 30.6, 41.1, 68.1, 70.7, 71.5, 72.3, 80.9, 126.7, 129.3, 134.0, 136.0, 137.2, 168.0, 168.5, 171.2. MS (ESI) m/z calculated for C_21_H_24_N_4_O_6_: 428.2; found: 429.2[M+H]^+^.

**Figure:**
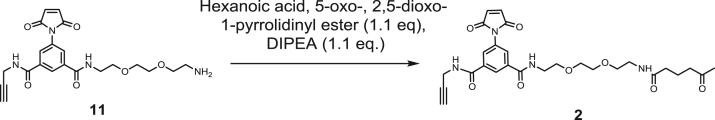

Synthesis of 5-(2,5-dioxo-2,5-dihydro-1H-pyrrol-1-yl)-N1-(2-(2-(2-(5-oxohexanamido)ethoxy)ethoxy)ethyl)-N3-(prop-2-yn-1-yl)isophthalamide 2A solution of compound 11 (1.0 g, 2.3 mmol) in DMF (2 mL) was treated 2,5-dioxopyrrolidin-1-yl 5-oxohexanoate (0.63 g, 2.8 mmol) and DIPEA (0.4 mL, 2.8 mmol). The resulting mixture was stirred at room temperature for 1 h followed by removal of solvent under reduced pressure. The resulting crude mixture was purified by reverse phase chromatography to yield 2 as a white solid (0.58 g, 47%). ^1^H NMR (400 MHz, Methanol-*d4*) δ 8.19 (s, 1H), 7.88 (s, 2H), 6.91 (s, 2H), 4.07 (s, 2H), 3.58-3.37 (m, 10H), 2.67 (s, 2H). 2.37 (m, 2H), 2.04 (m, 2H), 1.99 (s, 3H), 1.67 (m, 2H). ^13^C NMR (101 MHz, Methanol-*d4*) δ 19.6, 25.1, 28.8, 38.9, 39.7, 69.1, 69.2, 71.2, 79.3, 124.9, 127.8, 132.5, 134.4, 135.5, 166.4, 169.5, 174.2, 209.8. MS (ESI) m/z calculated for C_27_H_32_N_4_O_8_: 540.2; found: 541.2 [M+H]^+^.

### 2.3 Synthesis of SG3457

**Figure:**
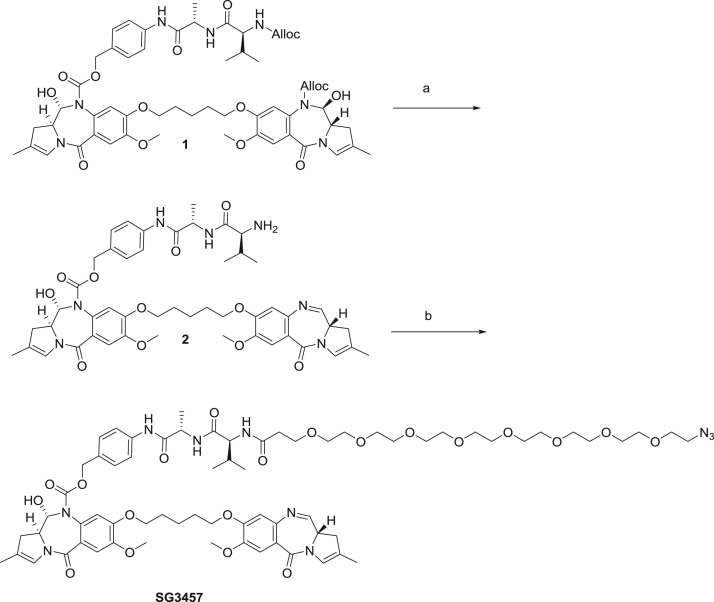

**Synthesis of SG3457.** (a) Pd(PPh_3_)_4_, pyrrolidine, DCM, (b) Azido-dPEG®_8_-acid, EDCI.HCl, 3% MeOH:CHCl_3_, 41%.

### 2.4 General information for the synthesis of SG3457

Reaction progress was monitored by thin-layer chromatography (TLC) using Merck Kieselgel 60 F254 silica gel, with fluorescent indicator on aluminium plates. Visualisation of TLC was achieved with UV light. Flash column chromatography was performed using Merck Kieselgel 60 F254 silica gel. Extraction and chromatography solvents were bought and used without further purification from Fisher Scientific, U.K. All chemicals were purchased from Aldrich, Lancaster or BDH. Azido-dPEG®_8_-acid was purchased from Quanta biodesign.

^1^H and ^13^C NMR spectra were obtained on a Bruker Avance 400 spectrometer. Coupling constants are quoted in hertz (Hz). Chemical shifts are recorded in parts per million (ppm) downfield from tetramethylsilane. Spin multiplicities are described as s (singlet), bs (broad singlet), d (doublet), t (triplet), q (quartet), p (pentuplet) and m (multiplet).

The LC/MS conditions were as follows:

*Method 1 (3 min run)*

The HPLC (Shimadzu Nexera®/Prominence® LCMS-2020) was run using a mobile phase of water containing 0.1% formic acid (A) and acetonitrile containing 0.1% formic acid (B). Gradient: 5% B held over 25 s, then increased from 5% B to 100% B over a 1 min 35 s’ period. The composition was held for 50 s at 100% B, then returned to 5% B in 5 s and held there for 5 s. The total duration of the gradient run was 3.0 min.

*Method 2 (15 min run)*
  The HPLC (Shimadzu Nexera®/Prominence® LCMS-2020) was run using a mobile phase of water containing 0.1% formic acid (A) and acetonitrile containing 0.1% formic acid (B). Gradient: 5% B held over 1.0 min, then increased from 5% B to 100% B over 9 min. The composition was held for 2 min at 100% B, then returned to 5% B in 10 s and held for 2 min 50 s. The total duration of the gradient run was 15.0 min.

Flow rate was 0.8 mL/min (for 3-min run) and 0.6 mL/min (for 15-min run). Detection was at 254 nm. Columns: Waters Acquity UPLC® BEH Shield RP18 1.7 µm 2.1 × 50 mm at 50 °C fitted with Waters Acquity UPLC® BEH Shield RP18 VanGuard Pre-column, 130 A, 1.7 µm, 2.1 mm x 5 mm (routine 3-min run); and ACE Excel 2 C18-AR, 2 µ, 3.0 × 100 mm fitted with Waters Acquity UPLC® BEH Shield RP18 VanGuard Pre-column, 130 A, 1.7 µm, 2.1 mm x 5 mm (15-min run).

The preparative HPLC conditions were as follows: Reverse-phase ultra-fast high-performance liquid chromatography (UFLC) was carried out on a Shimazdzu Prominence® machine using a Phenomenex® Gemini NX 5 µ C18 column (at 50 °C) 150 × 21.2 mm. Eluents used were solvent A (H_2_O with 0.1% formic acid) and solvent B (CH_3_CN with 0.1% formic acid). All UFLC experiments were performed with gradient conditions: Initial composition 13% B increased to 100% B over a 15 min period. The composition was held for 2 min at 100% B, then returned to 13% B in 0.1 min and held there for 2.9 min. The total duration of the gradient run was 20.0 min. Flow was 20.0 mL/min and detection was at 254 and 280 nm.

**(*****11S*****)-4-(2-(1-((1-amino-3-methyl-1-oxobutan-2-yl)amino)-1-oxopropan-2-yl)hydrazinyl)benzyl 11-hydroxy-7-methoxy-8-((5-((7-methoxy-2-methyl-5-oxo-5,11a-dihydro-1H-benzo[e]pyrrolo[1,2-a][1,4]diazepin-8-yl)oxy)pentyl)oxy)-2-methyl-5-oxo-11,11a-dihydro-1H-benzo[e]pyrrolo[1,2-a][1,4]diazepine-10(5H)-carboxylate (2).**

*Tetrakis***(**triphenylphosphine**)**palladium(0) (6.8 mg, 5.9 µmol, 0.06 eq.) was added to a solution of **1**^1^ (109 mg, 98 µmol, 1.0 eq.) and pyrrolidine (17.5 mg, 20.3 μl, 0.25 mmol, 2.5 eq.) in dry dichloromethane (5 mL) under an Argon atmosphere. The reaction was stirred for 1 h at room temperature diluted with dichloromethane and washed with saturated aqueous ammonium chloride solution (10 mL) and brine (10 mL). The organic phase was dried over magnesium sulphate, filtered and the dichloromethane removed by rotary evaporation under reduced pressure. The resulting product **2** was used without further purification. (spectroscopic data can be found in ref Tiberghien et al. [2])

**4-((29*****S*****,32*****S*****)-1-azido-29-isopropyl-32-methyl-27,30-dioxo-3,6,9,12,15,18,21,24-octaoxa-28,31-diazatritriacontan-33-amido)benzyl (11S,11a*****S*****)-11-hydroxy-7-methoxy-8-((5-(((*****S*****)-7-methoxy-2-methyl-5-oxo-5,11a-dihydro-1H-benzo[e]pyrrolo[1,2-a][1,4]diazepin-8-yl)oxy)pentyl)oxy)-2-methyl-5-oxo-11,11a-dihydro-1H-benzo[e]pyrrolo[1,2-a][1,4]diazepine-10(5H)-carboxylate (SG3457).**

1-ethyl-3-(3’-dimethylaminopropyl)carbodiimide hydrochloride (EDCI.HCl, 18.9 mg, 98 μmol, 1.0 eq.) was added to a solution of compound **2** (91 mg, 98 μmol, 1.0 eq.) and Azido-dPEG®_8_-acid (46 mg, 98 μmol, 1.0 eq.) 3% methanol in chloroform (2 mL). The reaction mixture was stirred at room temperature for 18 h under an argon atmosphere. LC/MS and TLC analysis (5% MeOH in DCM) indicated the presence of a small amount of starting material. Additional portions of Azido-dPEG®_8_-acid (9.2 mg, 19.7 μmol, 0.2 eq.) and EDCI.HCl (3.8 mg, 19.7 μmol, 0.2 eq.) were added and the reaction continued for a further hour. The reaction mixture was diluted with dichloromethane (10 mL) and washed sequentially with water (10 mL) and brine (10 mL). The organic phase was dried over magnesium sulphate, filtered, and dichloromethane removed by rotary evaporation under reduced pressure. The crude material was purified by column chromatography (4 to 11% MeOH/CHCl_3_) to give the product as an off white foam (55 mg, 41%) LC/MS, RT = 6.94 min (ES+) *m/z* (relative intensity) ([M+]^+.^1371, 25). [α]^22^_D_ = +340.8° (*c* = 1.0, CHCl_3_). 1H NMR (400 MHz, CDCl_3_) δ 8.82 (s, 1H), 8.87 (s, 1H), 7.68 – 7.58 (m, 2H), 7.5 (s, 1H), 7.35 – 7.4 (s, 1H), 7.43 – 7.1 (m, 4H), 6.82 (s, 1H), 6.74 (s, 1H), 6.68 (s, 1H), 6.48 (s, 1H), 5.77 – 5.73 (m, 1H), 5.35 – 5.25 and 4.8 – 4.7 (2 x m 2H), 4.65 – 4.59 (m, 2H), 4.3 – 4.19 (m, 2H), 4.14 – 4.0 (m, 2H), 3.91 (s, 3H), 3.87 (s, 3H), 3.84 – 3.79 (m, 2H), 3.68 – 3.6 (m, 32H), 3.41 – 3.41 (m, 2H), 3.25 – 3.15 (m, 1H), 3.05 – 2.85 (m, 2H), 2.70 – 2.55 (m, 2H), 2.50 – 2.44 (m, 2H), 2.26 – 2.15 (m, 1H), 1.93 – 1.8 (m, 4H), 1.84 (s, 3H), 1.77 (s, 3H), 1.7 – 1.51 (m, 2H), 1.45 – 1.33 (m, 3H), 1.0 – 0.75 (m, 6H).

### 2.5 General procedure for linker-antibody conjugation

Heterofunctional linker **1** was conjugated to desired antibody in multiple steps. First, antibodies were mildly reduced to generate free thiols by adding 50 mM TCEP solution to 5 mL of 3.6 mg/mL antibody solution in 10 mM PBS, pH 7.4, 1 mM EDTA. The resulting solution was gently mixed at 37 °C for 1 h. Reduced antibody was transferred to a slide-a-lyzer dialysis cassette (10 K MWCO) and dialyzed against PBS, 1 mM EDTA, pH 7.4, 4 °C for 24 h with several buffer changes. Reduced antibody was oxidized to reform internal disulfides by addition of dehydroascorbic acid (50 mM stock in DMSO, 20 eq.) followed by gentle mixing for 4 h at room temperature. To the oxidized antibody solution was then added a solution of heterofunctional linker 1 (10 mM, DMSO, 4 eq.) The resulting reaction mixture was briefly vortexed and further incubated for the desired amount of time followed by addition of N-acetyl cysteine (10 μL of a 100 mM solution in water, 50 eq) and further incubation for 15 min to quench unreacted maleimide. All conjugation reactions were performed at room temperature (22 °C) under ambient atmosphere.

### 2.6 General procedure for copper catalyzed click reaction

Catalyst cocktail was prepared in a separate vial containing 0.64 mL of water and a solution of CuSO_4_ (0.24 mL 100 mM) was added a solution of BTTAA ((4-{[bis-(1-tert-butyl-1H-[1,2,3]triazol-4-ylmethyl)-amino]-methyl}-[1,2,3]triazol-1-yl)-acetic acid) (2.4 mL, 50 mM). To the resulting deep blue solution was added sodium ascorbate (0.72 mL, 500 mM) and the mixture vortexed until the color disappeared. The final concentration of this cocktail was as follows: CuSO_4_= 6 mM, BTTAA = 30 mM, sodium ascorbate = 90 mM. In a separate tube containing a solution (10 mM PBS, pH 7.4) of antibody conjugated with linker 1 (8.5 mg/mL) was added the payload equipped with the azido group (10 mM, DMSO, 8 eq.). The final concentration of DMSO in the resultant solution was adjusted to 10% by adding free DMSO. To this solution was added the catalyst cocktail to attain a final concentration of CuSO4 as 1 mM. The resulting solution was gently mixed at room temperature for 5 h followed by purification using CHT (Ceramic hydroxyapatite) column.

### 2.7 General procedure for oxime ligation

In a tube containing a solution (10 mM PBS, pH 7.4) of antibody conjugated with linker 1 (8.3 mg/mL) was added the payload equipped with the aminoxy group (10 mM, DMSO, 8 eq.). The final concentration of DMSO in the resultant solution was adjusted to 10% by adding free DMSO. To this solution was added the m-phenylenediamine catalyst (1 M, pH = 7.2) to attain a final concentration of catalyst as 100 mM. The resulting solution was gently mixed at room temperature for 12 h followed by purification using CHT (Ceramic hydroxyapatite) column.

### 2.8 ADC characterization

**Fig. 1:**
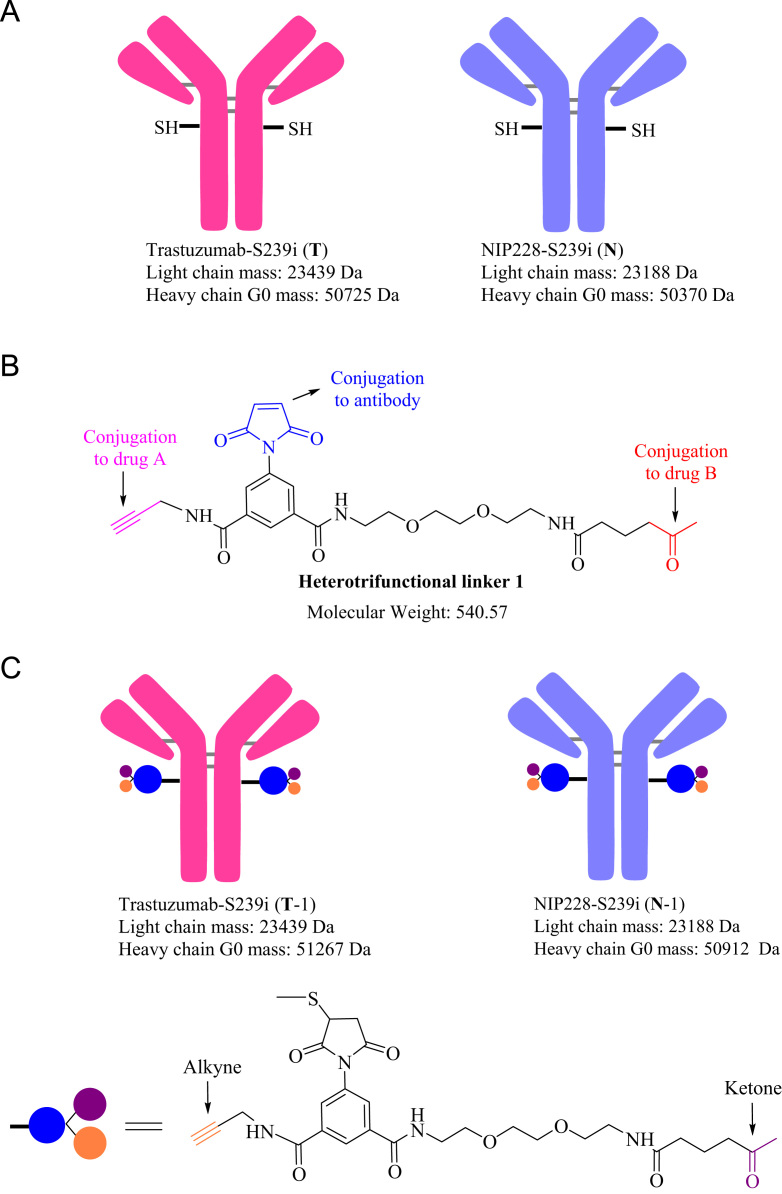

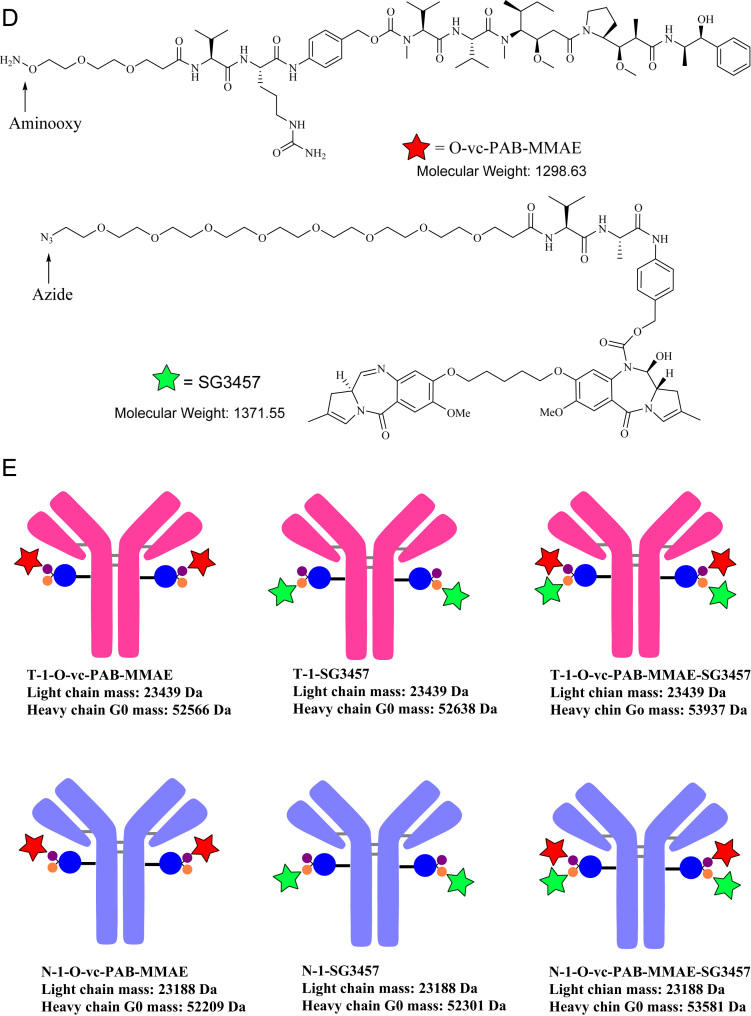

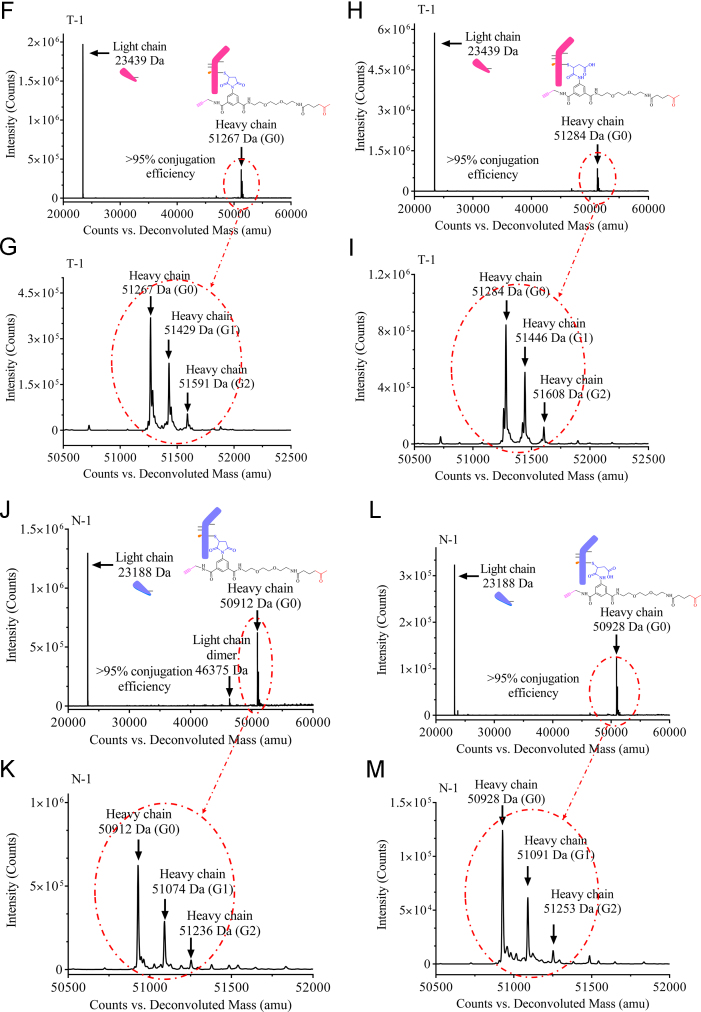

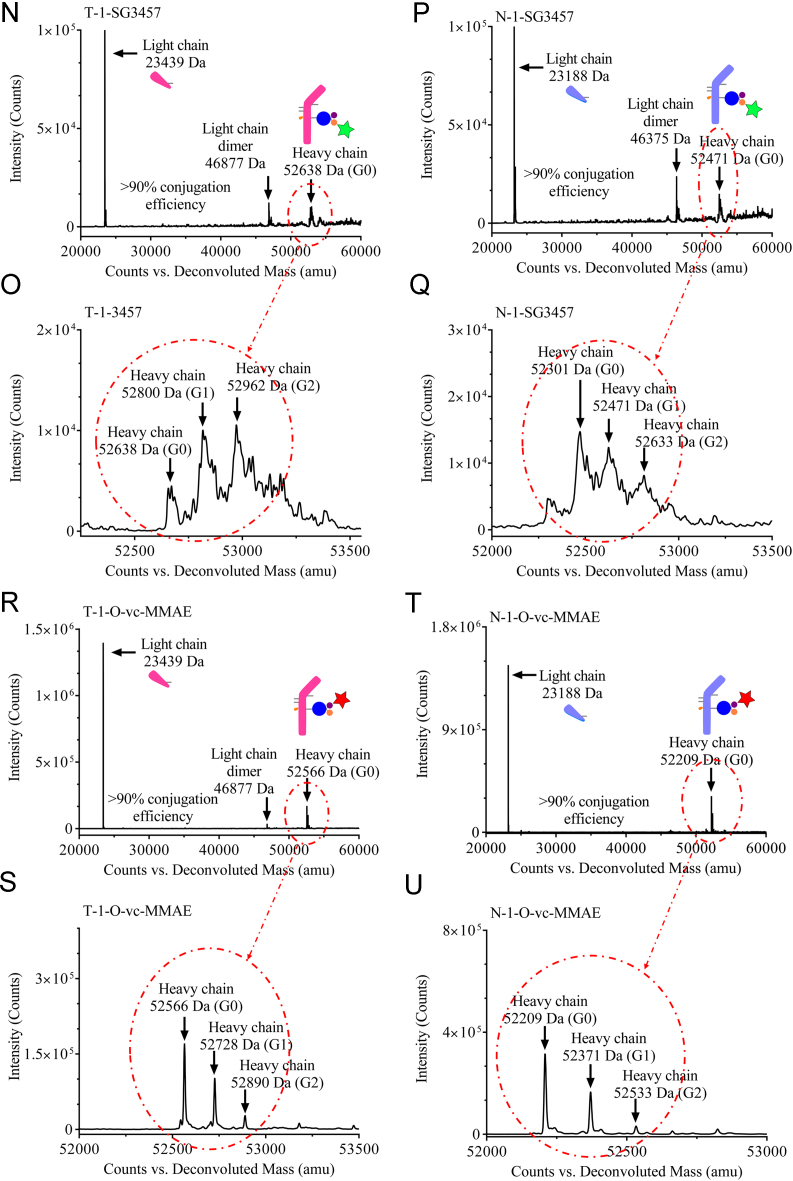

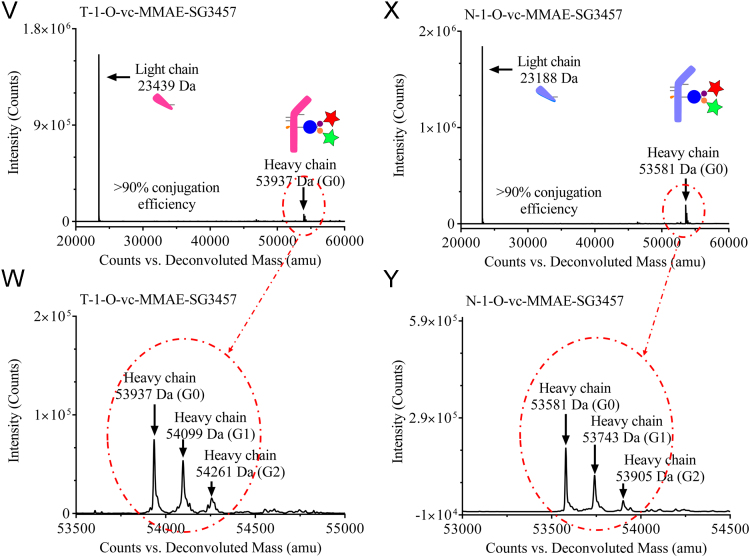
**(A)** Cartoon representation and masses of Trastuzumab-S239i (**T**) and NIP228-S239i (**N**) antibodies used for the preparation of the dual-warheads antibody-drug conjugates. (**B**) Structure and mass of the heterotrifunctional linker **1**. (**C**) Cartoon representation and masses of Trastuzumab-S239i (**T-1**) and NIP228-S239i (**N-1**) conjugated to the heterotrifunctional linker **1**. (**D**) Structure and masses of **O-vc-PAB-MME** (identified herein with a red star) and **SG3457** (identified herein with a green star). (**E**) Cartoon representation of the antibody-drug conjugates and their corresponding masses. (**F**) Reduced reverse phase liquid chromatography mass spectrometry (rLCMS) of non-hydrolyzed maleimide **T-1**. (**G**) Zoom of the non-hydrolyzed maleimide **T-1** heavy chain major glycoforms. (**H**) rLCMS of hydrolyzed maleimide **T-1**. (**I**) Zoom of the hydrolyzed maleimide **T-1** heavy chain major glycoforms. (**J**) rLCMS of non-hydrolyzed maleimide **N-1**. (**K**) Zoom of the non-hydrolyzed maleimide **N-1** heavy chain major glycoforms. (**L**) rLCMS of hydrolyzed maleimide **N-1**. (**M**) Zoom of the hydrolyzed maleimide **N-1** heavy chain major glycoforms. (**N**) rLCMS of **T-1-SG3457**. (**O**) rLCMS of **T-1-SG3457** major glycoforms. (**P**) rLCMS of **N-1-SG3457**. (**Q**) rLCMS of **N-1-SG3457** major glycoforms. (**R**) rLCMS of **T-1-O-vc-PAB-MMAE**. (**S**) rLCMS of **T-1-O-vc-PAB-MMAE** major glycoforms. (**T**) rLCMS of **N-1-O-vc-PAB-MMAE**. (**U**) rLCMS of **N-1-S O-vc-PAB-MMAE** major glycoforms. (**V**) rLCMS of **T-1-O-vc-PAB-MMAE-SG3457**. (**W**) rLCMS of **T-1-O-vc-PAB-MMAE-SG3457** major glycoforms. (**X**) rLCMS of **N-1-O-vc-PAB-MMAE-SG3457**. (**Y**) rLCMS of **N-1-S O-vc-PAB-MMAE-SG3457** major glycoforms.

**Fig. 2:**
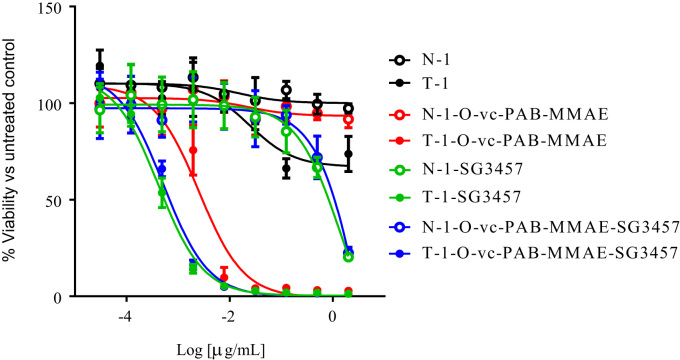
in vitro cytotoxicity of single and dual drug conjugates using Her2-expressing SKBR-3 breast cancer cell lines.

Reduced liquid chromatography mass spectrometry analysis (rLCMS), which was used to determine conjugation at the light or heavy chain and drug to antibody ratio (DAR), was performed on an Agilent 1290 series uHPLC coupled to an Agilent 6230 TOF. 2 μg of reduced antibodies or ADCs were loaded onto a Zorbax RRHD 300-Diphenyl (2.1 × 50 mm, 1.8 μm, Agilent) and eluted at a flow rate of 0.5 mL/min using a step gradient of 80% B after 2.1 min (mobile phase A: 0.1% Formic acid in water and mobile phase B: 0.1% Formic acid in acetonitrile). A positive time-of-flight MS scan was acquired, and data collection and processing were carried out using MassHunter software (Agilent). Conjugation efficiencies were calculated based on intensity of mass spectrometry signals of unconjugated vs conjugated (Fig. 1, Fig. 2).

### 2.9 Determination of in vitro cell viability

Human cancer cell lines SK-BR-3 and MDA-MB-453 were seeded into white polystyrene tissue-culture treated 96-well plates (Costar) at a density of 3000 cells/well in RPMI + 10% FBS (Invitrogen). On the following day, antibodies and ADCs were spiked into triplicate wells using an 8-point dose curve of 1:4 serial dilutions starting from 0.5 μg/mL. Cell viability was determined 6 days later using the Cell Titer-Glo Luminescent Cell Viability Assay kit (Promega) following the manufacturer׳s protocol. Luminescence was measured using an EnVision 2104 Multilabel Reader (Perkin Elmer). Cell viability was calculated as a percentage of control untreated cells.

### 2.10 Western blotting

SK-BR-3 were treated with antibody linker conjugates and ADCs at 0.1 µg/mL for 24 hours. Cell lysates were prepared with Laemmli SDS sample buffer (Boston Bioproducts) and were run on 4%–12% gradient NuPAGE Novex bis-tris gels (Invitrogen). After transfer, polyvinylidene difluoride (PVDF) membranes (Invitrogen) were blocked with 5% nonfat dry milk and 0.1% Tween 20 (Sigma) in Tris-buffered saline (TBS) (Amresco) and incubated overnight at 4 °C with antibodies to phospho-histone 2AX (H2AX), phospho-histone H3, total H2AX or total histone H3 (Cell Signaling Technology) or antibodies to glyceraldehyde-3-phosphage dehydrogenase (GAPDH) (Sigma). Membranes were washed in 0.1% Tween 20 in TBS and then incubated for 1 h in horseradish peroxidase-conjugated (HRP) secondary antibodies (Jackson Immunoresearch). After washing, protein bands were detected using SuperSignal West Pico Chemiluminescent substrate (Pierce/Thermo Scientific) and captured on an ImageQuant LAS 4000 mini instrument (GE Healthcare).
