# Supplementary material for: Characterization and in vitro data of antibody drug conjugates (ADCs) derived from heterotrifunctional linker designed for the site-specific preparation of dual ADCs
Source: Data Brief. 2018 Nov 6;21:2208–20. doi: 10.1016/j.dib.2018.11.005 (PMC6265423; doi:10.1016/j.dib.2018.11.005)
Supplement: Supplementary file 1 — Supplementary material [file mmc1.docx]

This work was supported by MedImmune, the global biologics R&D arm of AstaraZeneca. The authors are employee of MedImmune and may own stocks or stock options.
